# Optimizing automated external defibrillator deployment within the walking golden window for out-of-hospital cardiac arrest cases: a case study from a Chinese city

**DOI:** 10.3389/fpubh.2025.1649542

**Published:** 2025-08-26

**Authors:** Zhaohui Qin, Jia Li, Shuyao Zheng, Da Xu, Wei Zhang, Lu Lu, Xianliang Yan, Tie Xu, Ningjun Zhao, Yan Xu

**Affiliations:** ^1^The Second Clinical Medical School, Xuzhou Medical University, Xuzhou, China; ^2^Xuzhou Emergency Medical Center, Xuzhou, China

**Keywords:** automated external defibrillator, out-of-hospital cardiac arrest, deployment, geographical model, China, public access defibrillation

## Abstract

**Background:**

Irreversible brain injury may begin 4–6 min after the onset of out-of-hospital cardiac arrest (OHCA) if no cardiopulmonary resuscitation (CPR) is provided. This period is commonly referred to as the “golden window” in China. Based on the walking distance within this window, we proposed an improved public access defibrillation (PAD) deployment strategy to enhance automated external defibrillator (AED) efficiency in typical Chinese cities.

**Methods:**

This observational study used two datasets (an AED inventory and an OHCA registry) to assess the current effectiveness of AED deployment in the urban area of the Xuzhou city, Jiangsu Province. Using Geographic Information System (GIS) to determine the optimal AED placement distance based on the golden window walking-route distance. We also used python to simulate the improved model.

**Results:**

In the model, a total of 1,350 OHCAs and 1,238 AEDs were included and 78.4% of OHCAs occurred in the community. The AED coverage rate within 100 m was 7.93 and 7.33% based on the straight-line model and walking-route model. The proportion of OHCAs where an AED was accessible within the walking distance of the golden window accounted for 53.04% on average, with an average of 1.19 AEDs per case. The optimal deployment distance for AEDs to achieve maximum efficiency and approximate the standards of developed cities (Average = 1, Proportion = 40%) is computed to be 270–280 m in straight line. The simulation demonstration of the improved model shows that the benefit is significantly improved.

**Conclusion:**

Our model verified the current mismatch between AED deployment and OHCA cases in Xuzhou city. Based on this, we proposed an improved allocation model, which demonstrated the potential to optimize AED deployment more effectively. Furthermore, by integrating updated PAD strategies, our model can be further adapted to support drone-based AED delivery systems, offering a flexible and data-driven approach for future implementation.

## Introduction

Cardiac arrest (CA) is a critical health concern that severely affects human life. Among the various types of CAs, out-of-hospital cardiac arrests (OHCAs) have consistently exhibited high mortality rates over the years. According to emergency medical services (EMS) statistics from various regions across China, the incidence of out-of-hospital cardiac arrest (OHCA) is 97.1 cases per 100,000 population, with a survival to discharge rate of only 1.2% (1, 2). Globally, the incidence of OHCA ranges from 53 to 62 cases per 100,000 population, with an overall survival rate of 8.8%. Oceania has the highest reported survival rate (16.2%; 95% CI: 5.9–26.5%), followed by Europe (11.7%; 95% CI: 10.5–13.0%), North America (7.7%; 95% CI: 6.9–8.6%), and Asia (4.5%; 95% CI: 3.1–5.9%) (3). The low survival to discharge rate is primarily due to the failure of patients to receive timely EMS. The median EMS response time in China is approximately 12 min (4). According to the AHA guidelines, brain injury begins within 4–6 min after cardiac arrest if no cardiopulmonary resuscitation (CPR) is provided (5). We refer to this critical period as the “golden window.” When EMS response exceeds this timeframe, the patient’s chance of survival decreases significantly (6).

According to international guidelines, the use of an automated external defibrillator (AED) within the golden time can significantly improve survival outcomes in cases experiencing OHCA. AHA has promoted structured public access defibrillation (PAD) programs to allow nonmedical first responders and lay bystanders to use AEDs (7). These guidelines also recommend that AEDs should cover areas reachable within 1–2 min of brisk walking, which corresponds to an approximate Euclidean distance of 100 m (8–11).

Although studies indicate that the proportion of the Chinese population capable of accessing to CPR has increased to 17.0%, usage rates of AEDs by bystanders remain below 0.1% (1). As such, emergency care research has focused on ensuring that OHCA patients can access AEDs for defibrillation as quickly as possible. In Western countries, AEDs are typically placed in public spaces, such as restaurants, shopping malls, and schools (12–14). However, only recently have these countries started to consider AED placement in residential communities and other high incidence OHCA zones outside public spaces (15). At present, research on AED distribution in East Asia has been scarce such as Hong Kong and Japan (16, 17). Unfortunately, the majority of these studies have been conducted in developed regions, and due to economic limitations, their findings may be largely inapplicable to countries like China, where third-tier and lower-tier cities are predominant.

Recently, many researchers have developed mathematical and geographic models to improve the accessibility of AEDs, such as analyzing the differences between straight-line distance and walking-route distance (16, 18, 19). In urban environments, the straight-line model may underestimate the average distance and overestimate the coverage (16). For most cities in China, it can be assumed that an AED located within walking-route distance during the golden window offers effective emergency response. Furthermore, we can reasonably assume that when the probability of OHCA cases having access to an AED approaches that of developed East Asian cities (40%) (20, 21), the deployment can be considered to offer comparable emergency response effectiveness. If, on average, each OHCA case has access to one AED, the deployment can be considered economically efficient. Accordingly, we performed a simulation experiment based on a modeling approach.

This study focuses on the deployment of AEDs in the central urban area of Xuzhou, a city in China with a total population of approximately 9 million, including about 3.2 million residents in the urban core. By applying geographic modeling to historical OHCA data from the region, we aim to determine optimal AED deployment strategies tailored to the characteristics of such urban areas. This study also aimed to improve AED accessibility for OHCA patients by investigating the impact of urban environments on AED coverage. Ultimately, our results may provide a scientific basis for governmental bodies involved in the development of AED deployment strategies.

## Methods

### Data sources

This study is a secondary data analysis of OHCA cases reported by emergency medical centers in the Xuzhou city from Jan 2021 to Dec 2023. Descriptive analysis was conducted based on historical OHCA case data. As the city does not have a dedicated OHCA database, cases were selected through secondary screening of all cases received by the emergency medical centers. The dataset includes information on the location and time of OHCA events and was recorded by the Xuzhou Emergency Medical Center. Data are limited to the central urban areas of the city.

In this study, all AEDs analyzed were deployed by the city’s Health and Family Planning Commission in collaboration with Mindray, a medical technology company, and were freely available for use by all residents. The AED models used in this study were BeneHeart C1 and BeneHeart C2. These devices were accessible for use from 6:00 a.m. to 7:00 p.m. daily. Information regarding the AEDs, including their specifications and deployment locations, was obtained from Mindray’s official website.

### Geographical analysis of AEDs and OHCAs

To analyze the geographic information for both OHCA cases and AED locations, we utilized the Gaode Map coordinate system to determine the latitude and longitude of each location. Geographical data were then imported into ArcGIS software. Using the “Average Nearest Neighbor,” we calculated the distance between each OHCA location and the closest AED in meters. Additionally, we computed the average emergency rescue radius of the AEDs. City map data were obtained from the Bigemap GIS website.

### Geographic model by AEDs and OHCAs

Using Gaode Map, we identified the area reachable within a 3 min (half the golden 6 min for emergency care) walking-route distance from each patient location, and visualized these areas in ArcGIS. Using this as a constraint, we conducted an AED benefit analysis starting from a 100-meter radius around OHCA cases, increasing in 20-meter intervals by “Multiple Ring Buffer.” Based on this, we identified the closest optimal range—where the proportion of OHCA cases with access to an AED is approximately 40%, and the average number of accessible AEDs per case is around 1. To facilitate government planning, we also generated actual coverage maps at 50-meter intervals.

We also used Python 2.7 to simulate the placement of 100 AEDs before and after optimization. Using the optimal distance obtained from our model, we conducted a simulation. Taking one AED as the origin, we used Python to randomly generate 100 points, ensuring that each point has at least one neighboring point within the optimal distance. We then compared the emergency coverage area achieved by these simulated points with that of the original 100 nearest points prior to optimization.

## Results

### Geospatial data of urban AED locations and OHCA incidents

**Figure 1:**
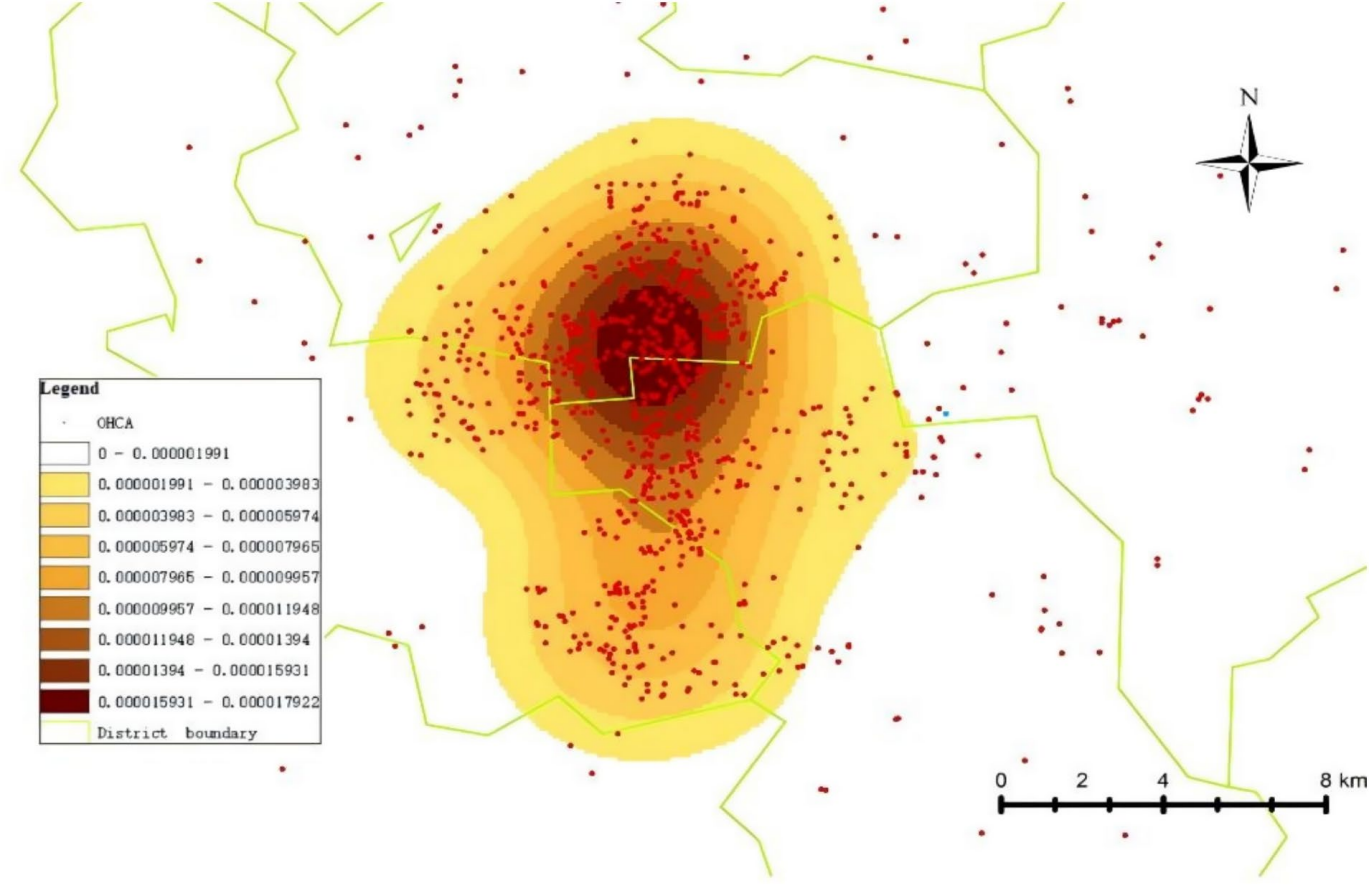
Geographic distribution of OHCA cases in the main urban area of the city (2021–2023).

From 2021 to 2023, a total of 1,350 cases of OHCA occurred in the central urban area of the city. As shown in Table 1, these cases were categorized based on their location, including communities (residential areas), schools, medical institutions, commercial streets, government offices, highways, enterprises, train stations, and tourist attractions. The majority of OHCA events occurred within communities, accounting for 1,058 cases (78.4%). A hot spot map showing the distribution of OHCA cases in the city’s central urban area from 2021 to 2023 is presented in Figure 1.

**Table 1:** Location of OHCA cases and AEDs.

Location type	OHCAs number (%)	AEDs number (%)
Community	1,058 (78.4)	352 (28.4)
School	26 (1.9)	204 (16.5)
Medical institution	49 (3.6)	349 (28.2)
Commercial street	93 (6.9)	28 (2.3)
Administration	27 (2)	211 (17.0)
Highway	26 (1.9)	0 (0)
Enterprise	22 (1.6)	16 (1.3)
Station	29 (2.2)	63 (5.1)
Tourist spot	20 (1.5)	15 (1.2)

**Figure 2:**
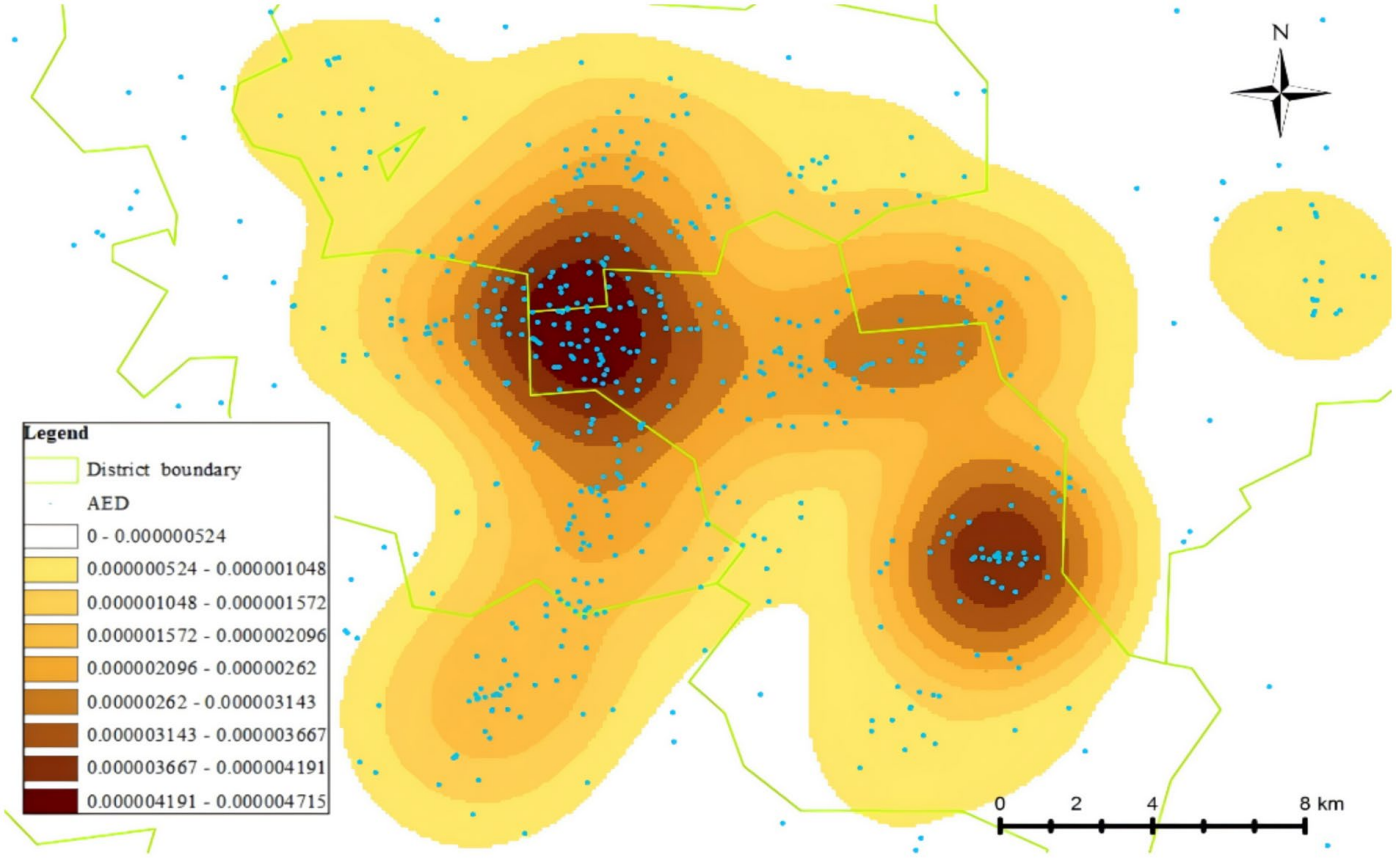
Geographic distribution map of AEDs in the main urban area of the city.

**Figure 3:**
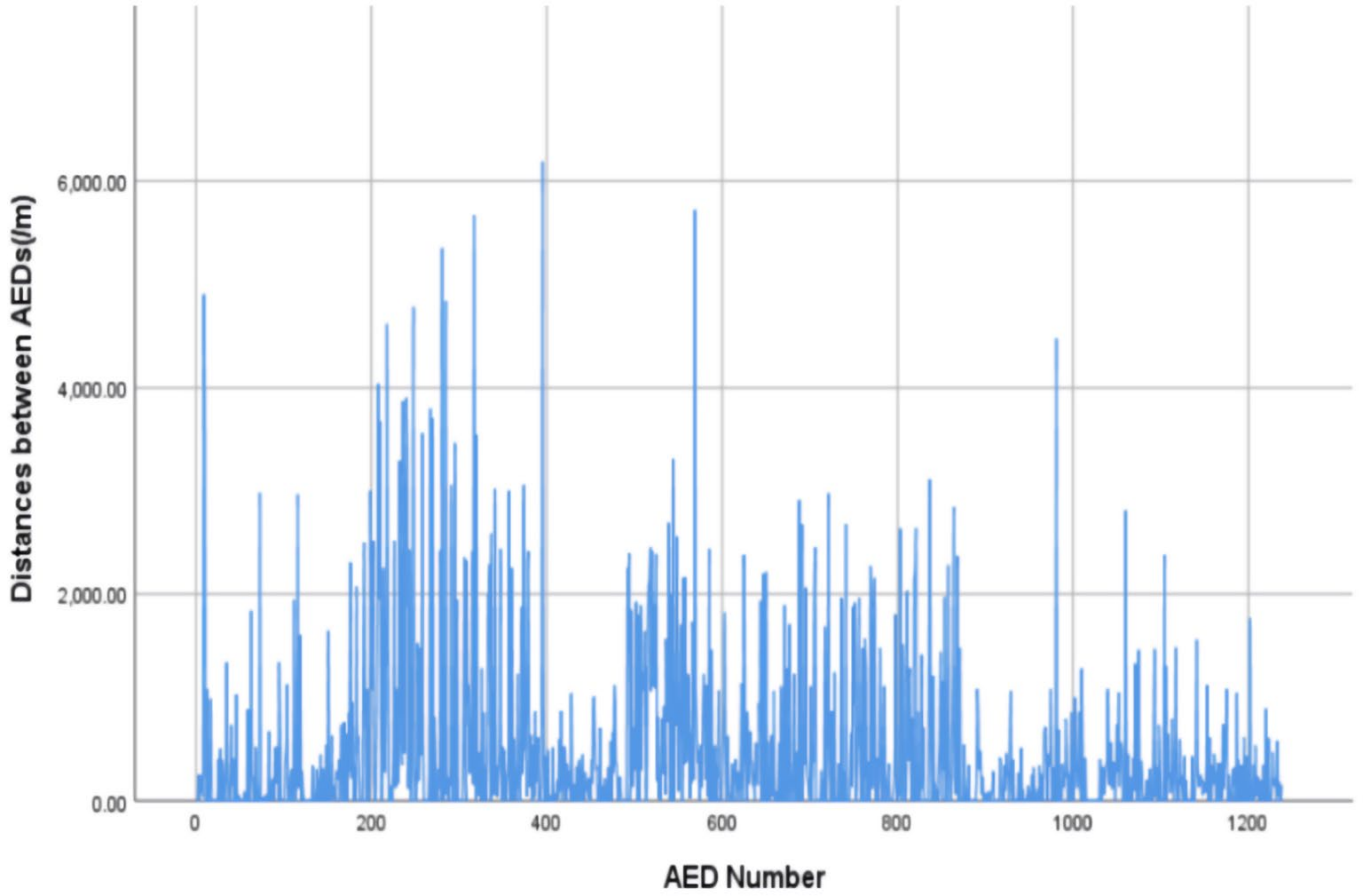
Histogram of the nearest straight-line distances between AEDs.

Among the 2,968 AEDs registered on the Mindray official website during the study period, 1,238 were located in the central urban area of the city. The per capita coverage was 3.87 AEDs per 10,000 people, with a density of 1.87 AEDs per square kilometer (area data for the central urban area sourced from official government publications). AEDs were installed in various locations, including schools, communities, shopping malls, train stations, enterprises, government office buildings, hospitals, and tourist attractions. Communities (352 units, 28.4%) and hospitals (349 units, 28.2%) had the highest number of AEDs (Table 1). The specific distribution pattern of AEDs is illustrated in Figure 2 while AEDs in the central urban area of the city were spaced relatively closely, indicating a high density of AEDs within this region. In fact, as shown in Figure 3, the straight-line distance between two adjacent AEDs was very uneven. The average distance between neighboring AEDs was 497.96 m (SD 838.17 m), with a median of 162.27 m (Table 2).

**Table 2:** Statistical scale of the nearest straight-line distances between AEDs.

Scale	Min	Q1 (25%)	Med	Q3 (75%)	Max	Avg	SD
Distance	0.00	0.00	162.27	546.85	6183.95	497.96	838.17

Min, minimum value; Q1, lower quartile; Med, median; Q3, upper quartile; Max, maximum value; Avg, average; SD, standard deviation.

The average straight-line distance from AED to OHCAs was 378.94 m (SD 351.74 m), with a median of 307.12 m. We observed a difference between the straight-line distance and walking-route distance. When converted to walking-route distance, the average walking-route distance was increased to 407.09 m (SD 442.30 m), with a median of 327.15 m (Table 3).

**Table 3:** Distance statistics for OHCA cases and AEDs (/m).

Distance	Min	Q1 (25%)	Med	Q3 (75%)	Max	Avg	SD
Straight-line	0.00	190.75	307.12	434.31	3993.28	378.94	351.74
Walking-route	0.00	195.06	327.15	450.00	5343.29	407.09	442.30

Min, minimum value; Q1, lower quartile; Med, median; Q3, upper quartile; Max, maximum value; Avg, average; SD, standard deviation.

### Distances to OHCAs and AEDs coverage geographic model

For AED coverage, our analysis was based on the commonly used 100 m threshold for both straight-line and walking-route distances. The results showed that 107 OHCA cases (7.93%) were within a 100 m straight-line distance from the nearest AED and on average, each patient has accessed to 0.11AED units. When converted to walking-route distance, 99 cases (7.33%) were within 100 m and the average AED availability per patient is 0.07 units. Additionally, based on the threshold of half the golden window (i.e., within 3 min), we found that 716 OHCA cases (53.04%) were reachable within the adapted walking-route distance, each patient has access to 1.12 AED units (Table 4).

**Table 4:** Relationship between distance to OHCA cases and number of AEDs within range.

Distance	100 m (W)	100 m	150 m	200 m	250 m	300 m	350 m	400 m	3 min (W)	Standard
AVG	0.07	0.11	0.24	0.43	0.67	1.00	1.38	1.82	1.19	1.00
%	7.33	7.93	16.87	26.67	36.74	47.63	59.26	68.00	53.04	40.00

Avg, average; %, proportion; W, walking-route.

**Figure 4:**
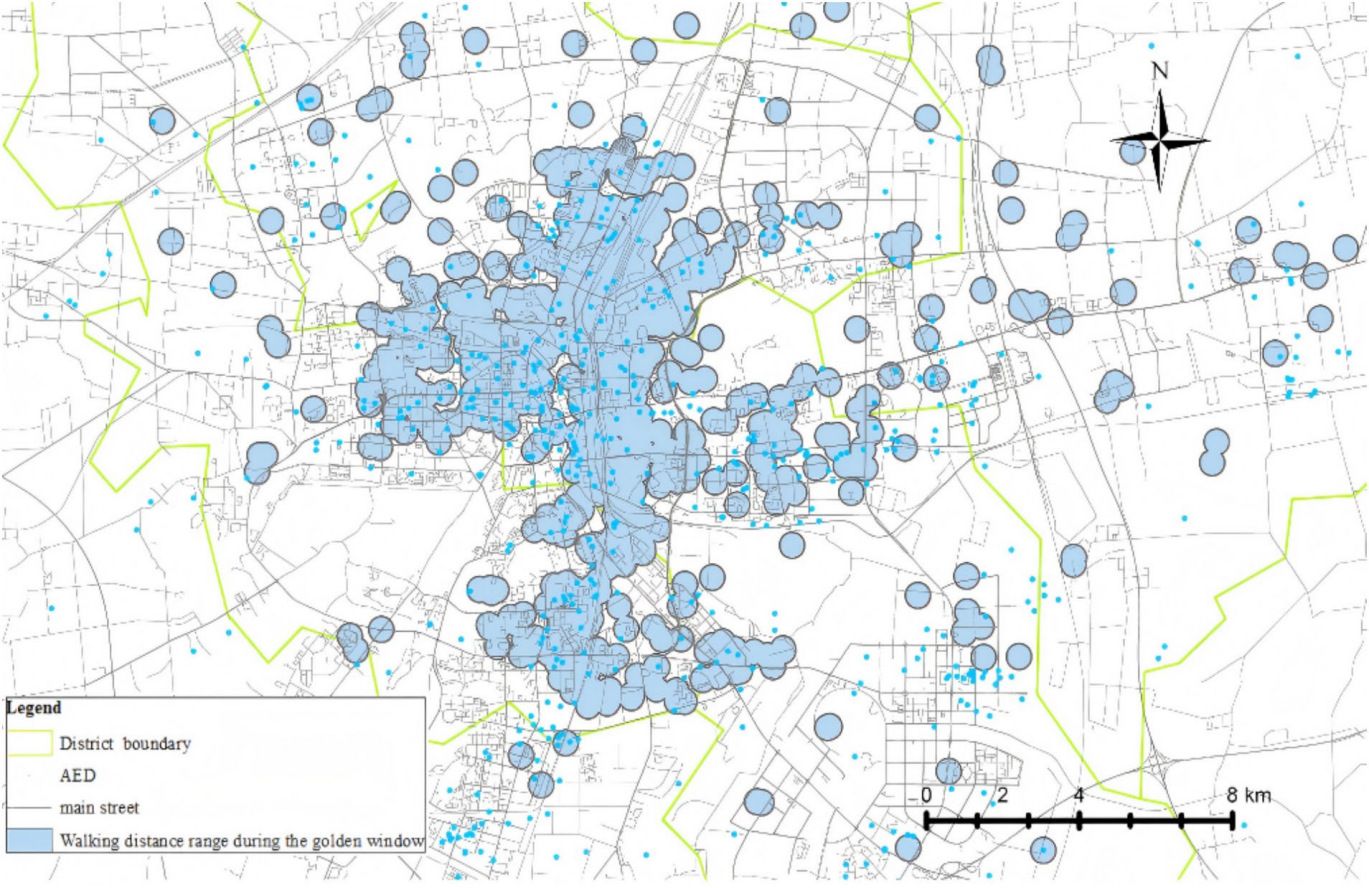
Three-minutes walking route coverage area for OHCA cases.

**Figure 5:**
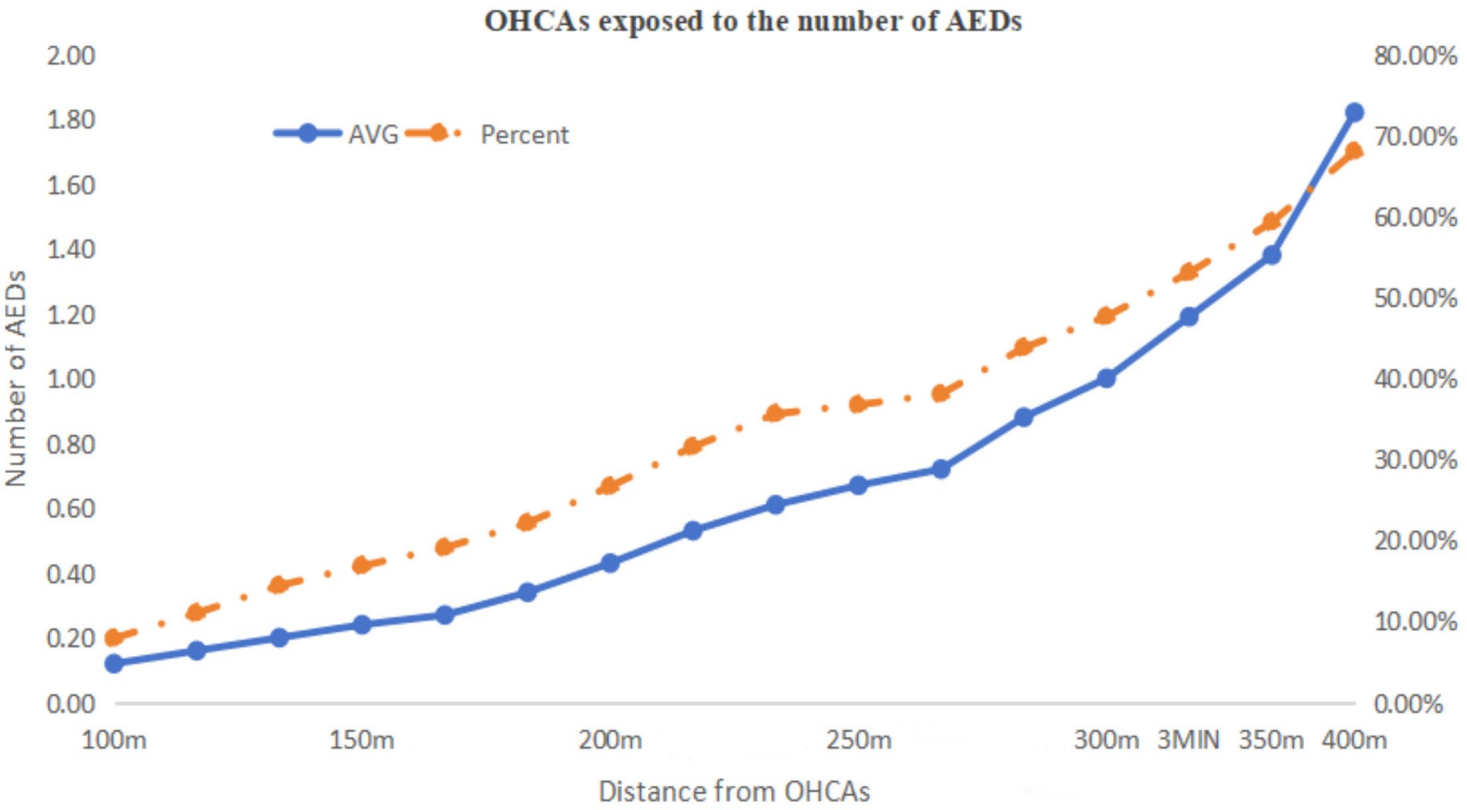
The relationship between distance to OHCA cases and AED accessibility for OHCA cases.

**Figure 6:**
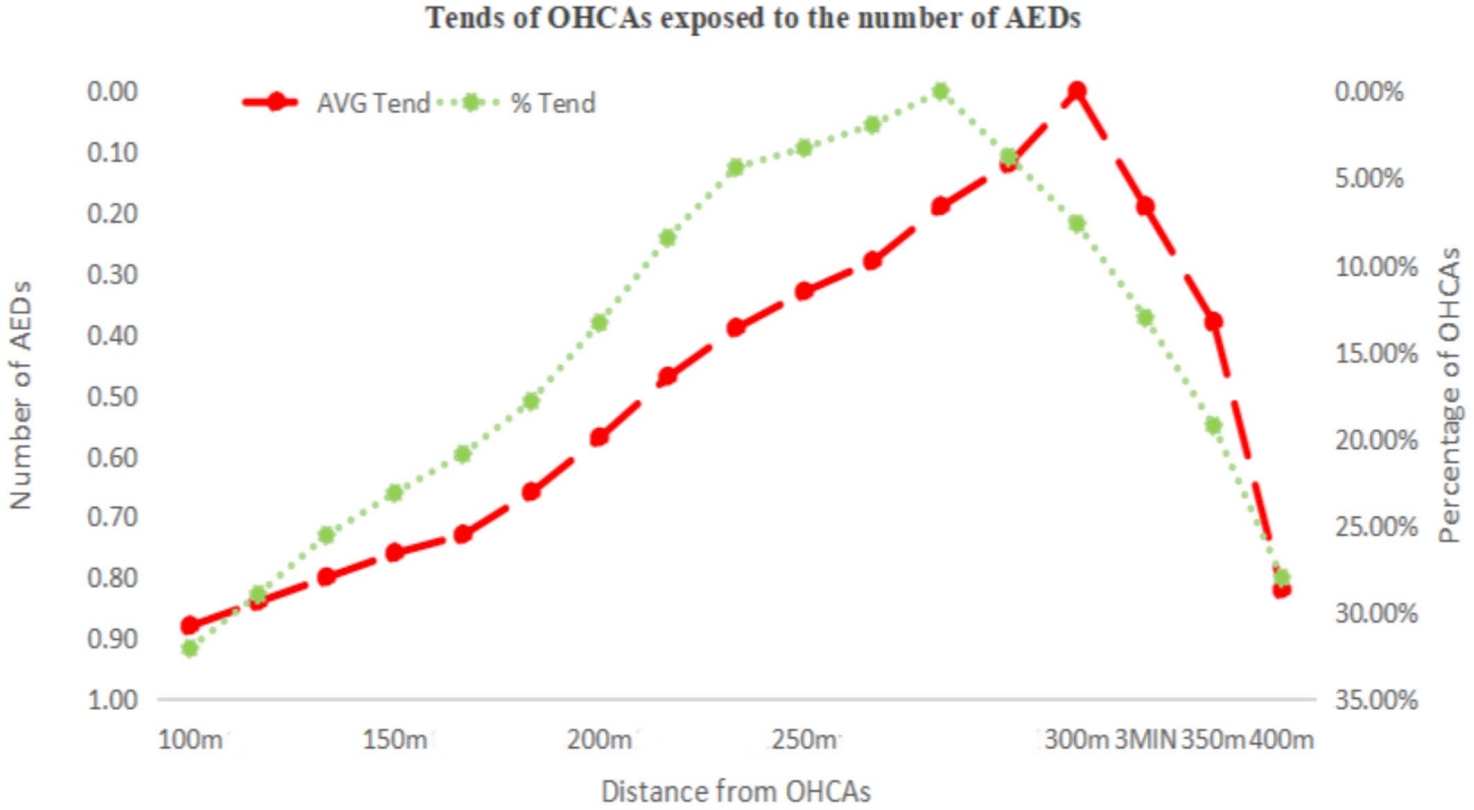
Trend between distance to OHCA cases and optimal emergency response benefit.

Figure 4 illustrates the area with emergency response effectiveness (3-min walking radius), which we used as the threshold. Based on this range and the data in Table 4, we plotted line charts showing the relationship between the average (AVG) and the proportion (%) versus linear distance (Figure 5), as well as trend charts depicting how both metrics approach optimal levels (AVG = 1, % = 40.00%) with increasing distance. We found that the values are closest to the optimal state when the distance is between 270 and 280 m (Figure 6).

**Figure 7:**
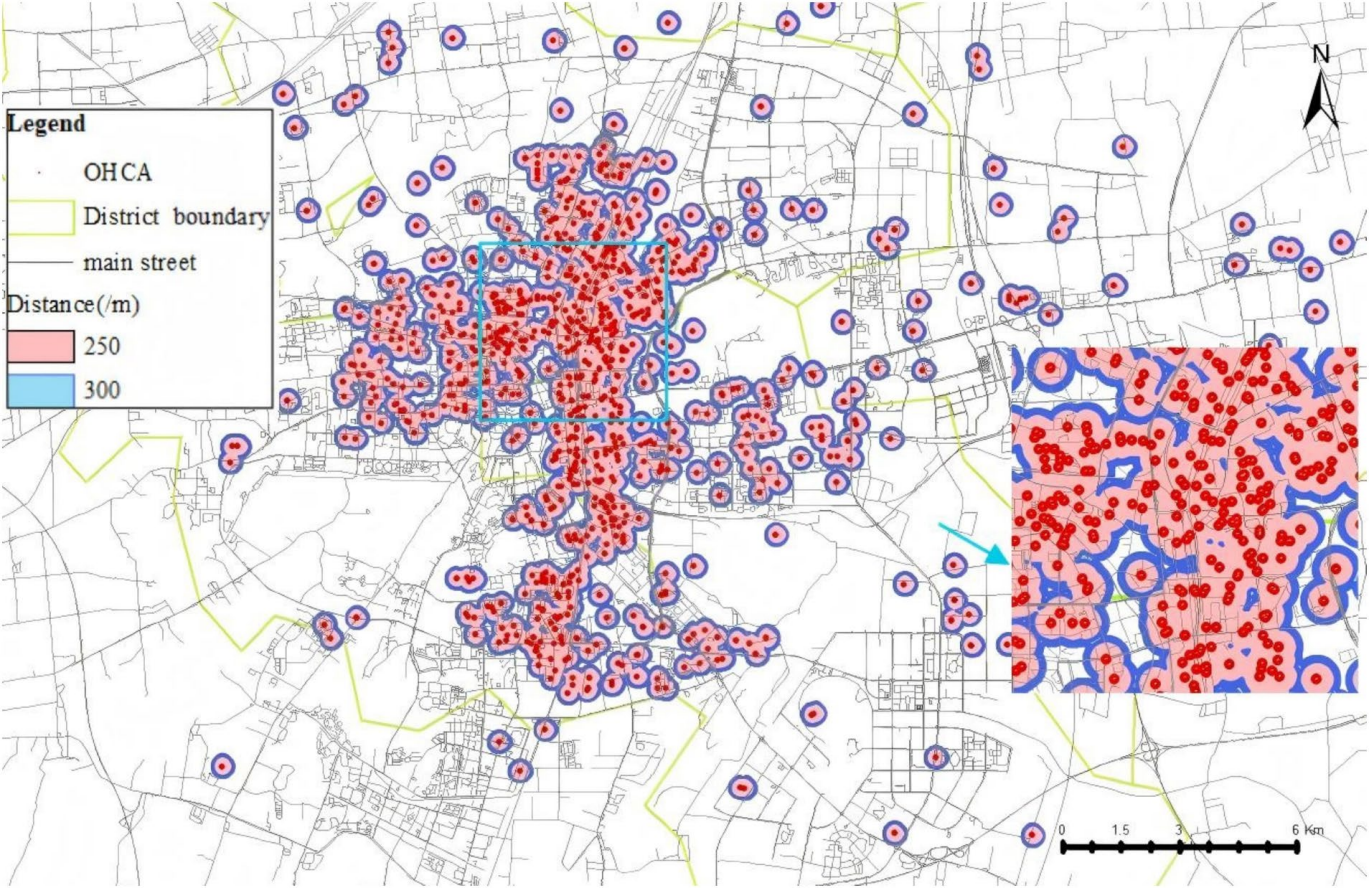
The geographical range of AED is optimally arranged according to OHCA cases.

**Figure 8:**
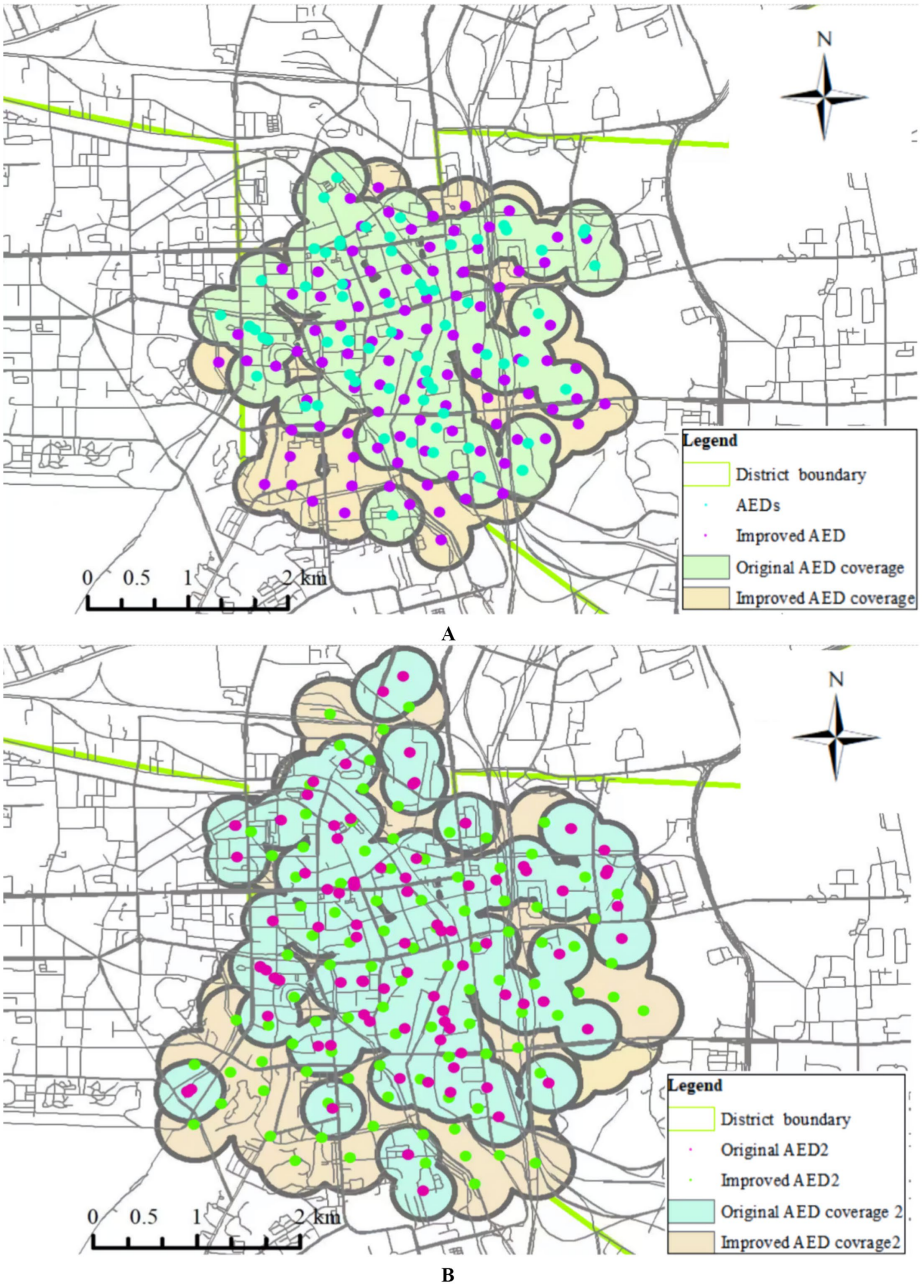
**(A)** First simulation test of 100 AEDs coverage before and after improvement. **(B)** Second simulation test of 100 AEDs coverage before and after improvement.

We conducted a geographic model simulation using a deployment distance of 250–300 m. The resulting coverage area that can rescue OHCAs is shown in Figure 7. Using this distance as the basis for AED placement, we found a significant improvement in the effective emergency response coverage. The effective coverage area increased from 6.31 km^2^before the simulation to 8.17 km^2^after the simulation. In another simulation, the area was increased from 8.75 km^2^ to 11.90km^2^ (Figure 8). Within the simulated region, the expected emergency response rate of AEDs could increase by above 30%.

## Discussion

Currently, most countries follow the guidelines for AED placement established by the European Resuscitation Council, which recommend situating AEDs in areas with high foot traffic, such as train stations, bus terminals, airports, sports facilities, and shopping centers (22–24). Our findings suggest that the AED deployment strategy in the studied city largely adheres to international conventions. The number of AEDs per square kilometer was comparable to that of Hong Kong, with the distribution of AEDs appearing to be relatively rational. However, the large SD (Standard Deviation) in placement distances indicates a concentration of AEDs in specific areas, primarily in city center administrative and commercial zones with dense populations. Conversely, industrial and agricultural areas, located farther from the city’s population centers, have fewer AEDs. The research has highlighted that cardiovascular disease mortality follows a social gradient (25). Individuals in underserved areas tend to experience prolonged durations of poor health, increased susceptibility to multiple diseases, and increased OHCA rates (26), which are further exacerbated by inequities in rural healthcare resources and longer EMS response times (27).

The average and median placement distances found in the current study suggest relatively high AED coverage rates within this city. In fact, the per capita AED coverage rate observed herein was higher than that in Hong Kong (1.94 per 10,000) and Busan (0.61 per 10,000) (28, 29) but was lower than that in developed cities, such as Copenhagen (9.2 per 10,000) and Toronto (6.68 per 10,000) (30, 31). However, the current AED emergency benefit and cost-effectiveness of the city are far lower than those in these developed areas.

The deployment of AEDs in the city needs to be optimized. To align with the standards of developed cities, studies have shown that walking-route distance measures are more accurate than straight-line distances (17). Deploying AEDs within a 3-min walking distance is more effective than using linear distance alone.

In this study, a deployment radius of approximately 270 m for AEDs was identified as optimal for improving emergency response efficiency. The median difference between walking distance and straight-line distance was 20 m, which is considerably smaller than the 90 m difference reported in similar studies. Given this minimal discrepancy, a deployment range of 250–300 m is recommended for policy consideration. In areas with complex terrain, the maximum deployment distance should not exceed 450 m. This recommendation aligns with findings reported by Remy in the Netherlands (32).

OHCA cases collected by the city’s emergency centers were predominantly located in communities and commercial streets. GIS-based spatial relationship models between AEDs and OHCA case locations indicate that the distribution of OHCA incidents largely overlaps with the AED locations, particularly in community and healthcare settings. AED placement in these areas facilitates access for individuals in nearby communities.

Notably, our data found a relatively low number of OHCA incidents occurring in public spaces, suggesting that AED placement strategies should focus more on residential communities, particularly in and around apartment buildings. This observation aligns with findings from international studies, such as those conducted in Japan (65.0% of OHCAs occurred at home) (33), the United States (69.8%) (34), and Victoria, Australia (70.2%) (35), all of which reported a predominance of OHCA events occurring in residential settings. Previously, the cost-effectiveness of placing AEDs in private homes had been considered low due to high costs and inefficiency (36). However, the increase in AED training and emergency response education in communities may significantly improve the cost-effectiveness of placing AEDs within these areas. Recent studies also support targeting communities as an effective means of expanding health coverage for families and workers, with strategies such as placing AEDs on each floor of residential buildings showing potential for enhancing emergency response outcomes (37–41).

Despite the relatively short distances to AEDs in most residential areas, the actual use of AEDs in emergencies was far lower than the model predictions. According to data from the Mindray website, only 65 AEDs (4.81%) had recorded usage, despite the occurrence of 1,350 OHCA cases. The number of patients who received immediate bystander CPR was also minimal, with almost no patient receiving AED-assisted resuscitation. This discrepancy may be due to a lack of public awareness and education on the use of AEDs (42). The duration between OHCA onset and AED usage by bystanders often exceeds the critical 3-min threshold (half of the 6 min golden window). Bystander response significantly influences OHCA survival rates. The duration from OHCA onset to EMS arrival involves several stages, including bystander recognition, emergency response activation, realizing the need for CPR and defibrillation, searching for an AED, and initiating rescue efforts. However, if bystanders are not aware of the need for an AED, the patient’s “chain of survival” might be interrupted (43). Moreover, limited 24-h accessibility of AEDs represents an important barrier to timely public access, particularly during holidays or nighttime hours when response from facility managers may be delayed or unavailable, or when certain areas are locked. This issue is especially evident in school settings (44).

To improve outcomes, focus should be placed not only on AED placement in high-risk areas but also on the entire survival chain, including early recognition, early bystander CPR, early defibrillation, and post-resuscitation care (45). A more comprehensive approach, including greater emphasis on emergency training and awareness, is necessary to support this chain and increase the effectiveness of AED deployment.

## Improvement

Given the high incidence and complex distribution of OHCA cases, establishing a unified and standardized OHCA database to facilitate more accurate tracking of epidemiological characteristics is imperative. This database should be developed based on the Utstein model, including core and optional elements such as the occurrence of OHCA, emergency dispatch data, patient demographics, treatment processes, and outcome prognoses (46–50). A standardized OHCA database would significantly enhance the scientific rigor and applicability of future studies, particularly cross-center research and comparative analyses, providing more robust and convincing evidence for clinical practice and policy-making.

Future AED deployment should not only consider the optimal deployment distance, but also be placed in the community as much as possible. Our findings also suggest the need for expanding AED training efforts beyond specific groups, such as students, to include the general community. Emphasis should also be placed on ensuring equitable access to resources and improving national healthcare systems to address disparities in healthcare access (51), which should start in urban areas and gradually extend to rural, industrial, and agricultural zones. Ensuring sufficient public training on CPR and AED usage could significantly reduce delays in the chain of survival.

The widespread use of smartphones could help addressed another challenge in enhancing AED access, that is, ensuring that bystanders can easily locate the nearest AED during an OHCA incident. Accordingly, developing effective mobile applications or integrating AED location systems into existing apps could guide users to the nearest device (52). Moreover, some regions are experimenting with “mobile AED” initiatives, placing AEDs on taxis and buses, and training drivers to become emergency volunteers. These drivers would be notified when an OHCA occurs nearby and would deliver the AED to the scene as quickly as possible and initiate CPR and defibrillation. Pilot programs are already underway in Chinese cities like Hangzhou and Chengdu in (53, 54). Such initiatives have the potential to expand AED coverage and improve utilization rates, ultimately enhancing public health outcomes. As technology continues to advance, new innovations offer new opportunities to improve AED access and usage (55, 56).

The Wolf Creek XVII report on PAD introduced the concept of mobile AED deployment, representing a departure from the traditional static PAD model (57). This approach has the potential to overcome well-documented barriers to early defibrillation and improve clinical outcomes. Among mobile strategies, the use of drones to deliver AEDs has attracted considerable attention and research interest in the academic community (58, 59). In this context, our geographic modeling approach may serve as a strategic enhancement by incorporating drone flight speeds to evaluate AED coverage within the critical response time. Compared to fixed AED placements, drone-based delivery offers the advantages of speed and precision; however, it also entails substantial financial investment. Our model could contribute to improving cost-effectiveness by optimizing deployment based on spatial–temporal accessibility, thereby enhancing the efficiency of emergency response systems.

## Limitations

First, the AED data used in this study were obtained from the city government’s registration records through the Mindray company’s official website. All AEDs analyzed were produced by this company and were of the same brand and model. Although Mindray AEDs cover most of Xuzhou’s urban area, devices from other brands purchased by government agencies or private entities were not included in our dataset, potentially leading to incomplete coverage data and underestimation of overall AED availability.

Second, the use of AEDs may be limited by time as not all AEDs are available for use at all times. For example, AEDs may be inaccessible at night due to the absence of custodial staff after working hours. This time-based accessibility issue can significantly affect the actual coverage of AEDs, with a marked reduction in AED availability during nighttime hours (44, 60, 61). This circadian disparity in public AED accessibility poses a critical challenge to establishing an equitable and effective OHCA response system. However, unattended placement of AEDs raises concerns about potential damage and theft. Strategies to balance accessibility and device security—such as insurance coverage for AEDs—remain to be explored (44). Meanwhile, the locations of OHCA incidents in this study were primarily recorded by EMS personnel accompanying ambulances. Hence, discrepancies may have been present between the recorded location and the actual site of the event, which could have also affected the true coverage of AEDs for OHCA cases.

Furthermore, our assumption were measured on the same horizontal plane. However, given that the city has many high-rise buildings, the actual distances between OHCA cases and AEDs could have been underestimated for AED located in or OHCA occurring on higher floors (62). To address this, future studies should explore three-dimensional modeling approaches, such as the Shortest Path Voronoi diagram method (63), which incorporates various factors, such as bridges, tunnels, and stairs that may affect walking speed, to create more precise digital models.

## Conclusion

Our study found a suboptimal spatial match between AED deployment and the distribution of OHCA cases in the central urban area of Xuzhou, indicating that current AED placement is not well aligned with actual emergency needs. The findings suggest that greater emphasis should be placed on community-based PAD within residential areas. Furthermore, our proposed geographic optimization model significantly improved PAD response efficiency in simulation analyses. This model not only enhances traditional AED deployment strategies but also offers a novel framework for evaluating and improving emerging mobile AED solutions, such as drone-based delivery systems. By integrating spatial accessibility with time-critical coverage, our model may contribute to improving both the effectiveness and cost-efficiency of future emergency response planning.

## Data Availability

The original contributions presented in the study are included in the article and/or supplementary material. The data that support the findings of this study are available upon reasonable request and with permission from the Municipal Health Commission of Xuzhou, Xuzhou Emergency Medical Center and relevant authorities. Requests to access the datasets should be directed to the corresponding author.
